# Cost-effectiveness of preventive case management for parents with a mental illness: a randomized controlled trial from three economic perspectives

**DOI:** 10.1186/s12913-016-1498-z

**Published:** 2016-07-07

**Authors:** Henny J. Wansink, Ruben M. W. A. Drost, Aggie T. G. Paulus, Dirk Ruwaard, Clemens M. H. Hosman, Jan M. A. M. Janssens, Silvia M. A. A. Evers

**Affiliations:** Context, Prevention Department of the Parnassia Group, Lijnbaan 4, The Hague, 2512 VA The Netherlands; Department of Health Services Research, School for Public Health and Primary Care (CAPHRI), Faculty of Health, Medicine and Life Sciences, Maastricht University, Duboisdomein 30, Maastricht, 6229 GT The Netherlands; Department of Clinical Psychology, Radboud University, Postbox 9104, Nijmegen, 6500 HE The Netherlands; Department of Health Promotion, School for Public Health and Primary Care (CAPHRI), Faculty of Health, Medicine and Life Sciences, Maastricht University, Peter Debeyeplein 1, Maastricht, 6229 HA The Netherlands; Department of Developmental Psychopathology, Radboud University, Postbox 9104, Nijmegen, 6500 HE The Netherlands; Trimbos, Netherlands Institute of Mental Health and Addiction, Da Costakade 45, Utrecht, 3521 VS The Netherlands

**Keywords:** Case management, Coordinated care, Prevention, Inter-sectoral costs and benefits, Cost-effectiveness analysis, Children of parents with a mental illness, Mental health promotion

## Abstract

**Background:**

The children of parents with a mental illness (COPMI) are at increased risk for developing costly psychiatric disorders because of multiple risk factors which threaten parenting quality and thereby child development. Preventive basic care management (PBCM) is an intervention aimed at reducing risk factors and addressing the needs of COPMI-families in different domains. The intervention may lead to financial consequences in the healthcare sector and in other sectors, also known as inter-sectoral costs and benefits (ICBs). The objective of this study was to assess the cost-effectiveness of PBCM from three perspectives: a narrow healthcare perspective, a social care perspective (including childcare costs) and a broad societal perspective (including all ICBs).

**Methods:**

Effects on parenting quality (as measured by the HOME) and costs during an 18-month period were studied in in a randomized controlled trial. Families received PBCM (*n* = 49) or care as usual (CAU) (*n* = 50). For all three perspectives, incremental cost-effectiveness ratios (ICERs) were calculated. Stochastic uncertainty in the data was dealt with using non-parametric bootstraps. Sensitivity analyses included calculating ICERs excluding cost outliers, and making an adjustment for baseline cost differences.

**Results:**

Parenting quality improved in the PBCM group and declined in the CAU group, and PBCM was shown to be more costly than CAU. ICERs differ from 461 Euros (healthcare perspective) to 215 Euros (social care perspective) to 175 Euros (societal perspective) per one point improvement on the HOME T-score. The results of the sensitivity analyses, based on complete cases and excluding cost outliers, support the finding that the ICER is lower when adopting a broader perspective. The subgroup analysis and the analysis with baseline adjustments resulted in higher ICERs.

**Conclusions:**

This study is the first economic evaluation of family-focused preventive basic care management for COPMI in psychiatric and family services. The effects of the chosen perspective on determining the cost-effectiveness of PBCM underscore the importance of economic studies of interdepartmental policies. Future studies focusing on the cost-effectiveness of programs like PBCM in other sites and studies with more power are encouraged as this may improve the quality of information used in supporting decision making.

**Trial registration:**

NTR2569, date of registration 2010-10-12.

**Electronic supplementary material:**

The online version of this article (doi:10.1186/s12913-016-1498-z) contains supplementary material, which is available to authorized users.

## Background

Children of parents with a mental illness (COPMI) have an increased risk of developing mental health disorders such as depression, anxiety disorders, personality disorders and alcohol dependence [1–3]. Across different studies, relative risks of 1.5 to 8.0 have been found [2, 4–6] for COPMI in comparison with children of parents without a mental illness. Apart from the burden this may pose on children and caregivers, COPMI put a substantial burden on youth mental health services and child health expenditures [7]. Case registers of the Dutch Youth Mental Health Services show that COPMI consume five times the amount of mental healthcare than do other children, and that they are overrepresented in clinical care [8]. Furthermore, COPMI use more costly specialized youth care and youth protection services [9, 10] than do other children. The emotional, social, and economic burden of mental illness has also led to growing awareness, among professionals worldwide, of the impact that mental illness has on patients’ families and children in particular [11]. It is estimated that more than half of the male and two-thirds of female patients have minor children [12]. Epidemiological studies in the Netherlands and Norway already show one out of six to one out of three children having a parent with a mental illness [13, 14].

Parental mental illness is often accompanied by many adversities, such as a history of being abused or neglected in childhood, poverty, divorce, isolation, and children having special needs or behavioral problems. In fact, it is the accumulation of such adversities that forms the greatest threat to parenting quality and healthy child development [3, 4]. Parenting quality is defined as the quality and quantity of stimulation and support available to a child in his/her home environment. This accumulation of adversities calls for preventive and proactive family support. Since families of COPMI have a variety of needs in different domains, interventions aimed at improving parenting quality should include a variety of services; accordingly, this requires a comprehensive coordinated approach. One such approach is preventive basic care management (PBCM).

PBCM is a preventive program targeting threats to parenting quality [15]. By assessing multiple risk factors for poor parenting and the needs of families in different domains, facilitating access to preventive services, tailoring services to assessed needs and coordinating psychiatric and preventive services, PBCM aims to support effective parenting by maintaining a good balance between the adversities, vulnerabilities, and strengths of parents. Ultimately PBCM aims thereby to promote the socio-emotional development of COPMI and to reduce the risk of developing behavioral problems. The effects of PBCM on parenting outcomes (parenting quality, parenting skills and parenting stress) were studied in an RCT [16]. Evidence was found that PBCM had a statistically significant positive effect on parenting skills (*η*^*2*^ = .055, *p* < 0.05). Significant effects on the quality of parenting, and the frequency and intensity of parenting stress were not found, although findings did suggest trends toward improved parenting quality (*η*^*2*^ = .026, *p* < 0.10) and reduced frequency and intensity of parenting stress (*η*^*2*^ = .029, *p* < 0.10 and *η2* = .011, *p* < 0.10).

Serving the needs of families of COPMI within the available financial resources is a major issue in health systems worldwide [17, 18]. Furthermore, within governmental health policies there is a growing emphasis on coherent, efficient and cost-effective health systems [19]. In addition to the effectiveness of preventive interventions, the outcomes of cost-effectiveness analyses (CEAs) are becoming more and more important within healthcare decision making [20, 21]. However, to our knowledge, no CEAs on COPMI interventions have yet been performed [22, 23]. Since one of the aims of PBCM is to improve parenting quality and prevent child behavioral problems, it might diminish the need for costly services in the long run. Other studies on preventive parenting programs for vulnerable families (not specifically designed for families of COPMI) have shown long-term economic benefits. For example, Karoly and colleagues [24] reported governmental savings of up to $18,000 for the home visitation program Nurse-Family Partnership, related to better maternal and children’s health and effects on the life course such as maternal income, youth criminality and substance abuse. However, short-term benefits, e.g. fewer emergency room visits and better child development, could potentially already outweigh costs. By creating customized, efficient and optimized basic care packages for families, PBCM may lead to a reduction in costs by reducing overlap among services, which means PBCM is potentially already cost-effective in the short run.

The services which COPMI may encounter are widespread and include both services within the healthcare sector and services in other sectors, such as social (child) care, the educational sector and the criminal justice system. For example, the higher risk of academic underachievement, when borne out, may result in the need for special educational services, and alcohol misuse may result in police contact and arrests [4, 25]. Accordingly, although interventions may present financial expenses in the healthcare sector, considerable costs or benefits (i.e. cost savings) can be expected in other sectors. These are known collectively as inter-sectoral costs and benefits (ICBs). Drost et al. [26] identified over seventy different ICBs which can be included in health-related economic evaluations, depending on the type of intervention and the population of the program under study. Including ICBs within a CEA might affect the outcome of an evaluation, which, in turn, can affect decision making on interventions.

The aim of this study was two-fold. First, the study examined the costs and cost-effectiveness of PBCM in comparison with care as usual (CAU) - i.e. basic information about available COPMI-interventions, such as consultation and COPMI groups along with psychiatric treatment. A second aim of this study was to answer the question whether a shift from a narrow (healthcare) perspective to broader perspectives, in which either childcare costs (social care perspective) or childcare costs and other ICBs (societal perspective) were included, results in a change in the cost-effectiveness of PBCM.

## Methods

### Trial design

In a randomized controlled trial (RCT), participants were randomized to either the PBCM condition or the control condition [16]. Participants in the PBCM condition received preventive service coordination, while participants in the control condition received information about COPMI-interventions and had the opportunity to make use of COPMI consultations and COPMI support groups in addition to psychiatric treatment (CAU). The time horizon of the study was eighteen months. Data on the quality of parenting and costs were recorded at baseline (T0) and after nine (T1) and eighteen months (T2). The CEAs in this study were conducted from three perspectives: a) the healthcare perspective, which included costs for health and child/family support services, b) the social care perspective, which also included costs for childcare and c) the societal perspective, which was the most comprehensive and included all measured use of services, including ICBs within the educational sector, the criminal justice system and services for debt restructuring. All analyses included intervention costs.

### Participants

Participants were outpatients of a community mental health institute located in the urban, western part of the Netherlands. Patients with longstanding psychiatric problems and an accumulation of risk factors for poor parenting were selected. Inclusion criteria were: being treated for a psychiatric disorder, being a caregiver for a child aged between three and ten years of age, the parents being interested in PBCM, and the family being exposed to three or more of a list of sixteen risk factors for poor parenting. This list (see Table 1) was based on a literature review on the impact of parental mental illness on parenting quality, and on risk and protective factors for poor parenting, child abuse and neglect [15]. The age was restricted to the phase of life of the primary school age so that the group was more homogeneous. In order to study preventive effects in children, children with a mental health diagnosis (e.g. ADHD, or conduct disorder) were excluded. Other exclusion criteria included an expected duration of less than three months for further therapy, living outside the catchment area and previous help utilizing PBCM. Recruitment took place between September 2010 and April 2012; the last follow-up was between March 2012 and November 2013.

**Table 1:** Risk factors for poor parenting

1. single parenthood 2. little support from spouse 3. little network support 4. relational problems 5. partner with mental health problems 6. children with poor health/handicaps/difficult temperament 7. changes in family structure/housing 8. two or more life events in the past two years 9. housing problems 10. poverty or debts 11. parents having been abused as a child 12. severe psychiatric symptoms 13. low compliance with psychiatric treatment 14. impulse control problems 15. alcohol or drug problems 16. low intelligence	

### Interventions

Using a family-focused strength-oriented rehabilitation model, the focus was on strengthening positive parenting and providing community and network support [15, 27]. The PBCM intervention consisted of five steps: 1) the enrolment procedures, in which families were referred by the parent’s therapist, 2) a systematic assessment of the strengths and vulnerabilities regarding parenting and children’s development based on information from parents, children, school, therapists, and other services involved, 3) the design of an integrated preventive plan for tailored preventive care, which was discussed in a meeting with the parents and the services involved, 4) linking families to and coordinating services for childcare for young children, clubs for older children, community health services, services for debt restructuring and financial resources, and, finally 5) PBCM monitored the implementation of the plan and evaluated effects in regular meetings with parents and services. Every family had an own tailored plan, and a personal PBCM coordinator, who monitored whether indicated services were provided. Fidelity was systematically supervised in meetings with colleague-coordinators. The PBCM program ended when parenting and the children’s development were sufficient according to the PBCM coordinator and the continuity of the necessary services over a longer period was secured. Further information on the PBCM intervention can be found elsewhere [15].

In the control condition, all parents received a brochure about the impact of parental problems on children and information about available services, such as free consultations by a COPMI-expert or COPMI groups for parents and children in which they can exchange experiences and learn about coping with the challenges of living with the parental illness. Participation was optional. Parents could refer themselves or their children by calling the COPMI team.

### Outcome measure

The primary outcome measure was quality of parenting. This was measured using the Home Observation for Measurement of the Environment (HOME) Inventory [28, 29]. The HOME is an instrument used widely and internationally to measure the quality and quantity of stimulation and support available to a child in the home environment. This instrument measures the availability and impact of objects, events and interactions with parents and covers four dimensions, namely responsiveness, learning materials, stimulation, and harsh parenting. The HOME has been used worldwide in studies in different cultures, sometimes adapted to local child rearing beliefs and practices. These studies showed consistent relations between most items and children’s adaptive functioning [30]. We used the ‘Infant-Toddler’, ‘Early Childhood’, ‘Middle Childhood’ and ‘Early Adolescent’ versions of Vedder, Eldering and Bradley [31], which was used in studies with ethnic minorities in the Netherlands. Items and content differ for different age groups. Items were scored as binary (yes/no) by a trained interviewer. The score was based on observations and a semi-structured interview with the parent and focal child during a home visit of one hour (in Dutch, Turkish or Moroccan). Following the recommendation in the HOME manual [28], three interviewers were trained *in vivo* by the first author. We reached an inter-observer agreement of 96 % (i.e. the percentage of items that both observers scored the same in a joint observation).

Furthermore, several sample characteristics were assessed at T0. These included primary patient (mother and/or father), family structure (single-, two-parent family), diagnosis and disease progression of parent(s) (depressive and anxiety disorders, other Axis I disorders, personality disorders, comorbidity, severity of illness, chronic course of illness), ethnicity (Dutch, Moroccan, Turkish, Surinamese, Netherlands Antilles, other), children (number of children, age and gender of index child), number of risk factors and receiving social benefits (yes/no).

### Resource usage and costing

Costs were related to running PBCM or CAU (intervention costs) and to utilization of services. Costs were measured irrespective of who bears them and were indexed (in Euros) for the reference year 2012 using price indices from Statistics Netherlands [32]. Cost prices used for calculation can be obtained via supplementary material which is published online (Additional file 1).

#### Intervention costs

Intervention costs were calculated based on the average time spent by human resources needed to execute PBCM or CAU. The measurement of PBCM intervention costs was based on the time investment of the PBCM coordinator, plus the time investment by other professionals in the meetings. Information on the time invested in PBCM was retrieved from the medical records, counting all telephone calls, reported e-mail exchanges, home visits, face-to-face contact of the PBCM coordinator with parents or the family, and coordination meetings. Time spent by the coordinator on telephone calls and e-mails was valued at 23.90 Euros per contact. Series of several telephone calls or mails (three or more) were valued at 95.61 Euros, face-to-face contacts were valued at 119.51 Euros, home visits by PBCM including traveling time at 191.22 Euros and coordination meetings were valued at 191.22 Euros. The price rate of PBCM is the tariff as billed by the organization for integral costs, which includes gross salary costs plus overhead. We used one standard tariff for professionals for participating in the coordination meetings, namely 95.61 Euros.

The costs of the control intervention included optional participation in consultation and COPMI groups. Cost units for COPMI were the number of consultations as reported in the medical records (95.61 Euros) and participation in the COPMI groups by parents or children (350 Euros). Costs for psychiatric treatment are included in the healthcare service costs (see below).

#### Costs related to utilization of services

Costs related to the family’s utilization of services (healthcare costs, childcare costs, and other inter-sectoral costs) were measured by interviewing the parents, using a study-specific family support questionnaire (Dutch Services and Support Questionnaire, Vragenlijst Hulp en Ondersteuning, VHO). The VHO was based on the Trimbos/iMTA questionnaire for Costs associated with Psychiatric Illness (TiC-P) [33, 34], with an appended list of services from the PBCM manual [27]. The questionnaire was tested on five families and adapted to make it feasible in practice. Within the questions, we used a three-month time frame for highly frequent, inexpensive services, such as childcare services, and a six-month time frame for less frequent, highly expensive services, such as hospital admissions. The total service costs for each family were estimated by multiplying the quantity of each type of resource with its relevant cost price [35].

#### Health service costs

Health service costs included costs related to the use of mental healthcare, other primary and secondary care, youth care, such as youth care agencies and preventive family support. Most costs were calculated by multiplying the units (contacts, sessions, hours) with the standard cost prices as noted in the Dutch guidelines for health economic research and the manual of the iMTA questionnaire on intensive youth care [36, 37]. When these sources did not report prices for specific services, cost prices were drawn from reports of the Dutch Healthcare Authority and the National Health Tariffs Act or the Netherlands Youth Institute [38, 39]. When these reports did not provide cost prices for measured services, costs were estimated based on equivalent services for which cost prices were available.

#### Childcare costs

Childcare included day care (professional childcare) and babysitter (informal childcare). Cost prices for professional and informal childcare were drawn from the Dutch guidelines for health economic research [37].

#### Inter-sectoral costs

In addition to childcare services, other ICBs were measured. These included services in the educational sector, such as costs for special education, services in the criminal justice sector, such as costs for court proceedings, police services, and costs for debt restructuring services. These were calculated by multiplying the units (contacts, sessions, hours) with the prices provided by a Dutch manual for ICBs [40]. When the manual did not provide the required cost prices, these cost prices were estimated based on valuation techniques described in the manual or, if available, drawn from the manual of the iMTA questionnaire on intensive youth care [36].

### Randomization

After having given written informed consent, ninety-nine families were randomized on a 50–50 ratio, by drawing an envelope from a container; the envelopes contained either information about the PBCM condition or information about the control condition. After randomization, 49 families were assigned to the PBCM condition and 50 were assigned to the control condition by the researcher.

### Data preparation for analysis

Missing values and invalid scores of the items of the HOME and VHO were checked with the interviewer. Of the entered data, 10 % were double scored and checked for differences. Outliers and missing values in the total scores on the HOME were analyzed using the Missing Values Analysis in SPSS. Less than 5 % of the items of the HOME were missing. No outliers were found. Missing items of the HOME were imputed with the mean of the scores at T0, T1 and T2. Missing assessments of the HOME at T1 and T2 were imputed using the expectation maximization technique (EM) in SPSS. Because of differences in content and number of items in each age version of the HOME, we calculated standardized T-scores, range 0–100 and SD = 10, as suggested by Bradley (2009, February 12, personal communication) and De Beurs [41]. A higher T-score means better parenting quality.

If costing data were missing for T1 or T2, the mean costs of the other two measures (T0 and T1 or T2) for that family were imputed. If a family dropped out after baseline, the mean costs of the total group at T1 and T2 were imputed. Subsequently, measured costs were extrapolated [42]. To cover the period of nine months, costs were extrapolated by multiplying the costs related to highly frequent inexpensive services times three and the costs related to less frequent, highly expensive services times 1.5. Extrapolated costs for services measured at T1 and T2 were aggregated to cover the whole follow-up period of eighteen months, which were then used for the analyses.

### Analyses

Descriptive statistics were used to describe the characteristics of the sample at baseline. Differences between the groups were assessed using t-tests for continuous variables and chi-square tests for discrete variables in SPSS. From all three perspectives, for both conditions the costs were significantly tailed to the right (*p* < 0.01); skewness scores for the control and intervention condition were respectively 2.46 and 1.69 (healthcare perspective), 1.93 and 1.20 (social care perspective), and 1.67 and 1.05 (societal perspective). Skewed data is common among costing studies [43]. To determine the cost-effectiveness of PBCM, incremental cost-effectiveness ratios (ICERs) were calculated from all three perspectives (healthcare, social care and societal). Results are presented in cost-effectiveness planes and cost-effectiveness acceptability curves (CEACs) [35, 44].

Box 1 The incremental cost-effectiveness ratio, the cost-effectiveness plane and the cost-effectiveness acceptability curve.

**Table:** 

The ICER is a ratio comparing the additional costs and effects in the experimental intervention with the control intervention. ICERs were calculated using the formula: $ICER=\frac{\left({C}_i\ \hbox{--}\ {C}_c\right)}{\left({E}_i\ \hbox{--}\ {E}_c\right)}$ In this study, C represents the average total costs per family during the whole follow-up period of eighteen months, and E represents the mean difference between the HOME score at T2 and the HOME score at T0 in the PBCM condition (subscript i) and control condition (subscript c). Stochastic uncertainty in the data was dealt with using non-parametric bootstraps. By using the bootstrapping technique in Excel, the original sample was re-sampled, which resulted in 5000 simulated ICERs per scenario. These were plotted in cost-effectiveness planes (Fig. 2a,b,c). These planes provide a visual representation on the probability of PBCM being cost-effective in comparison with the control condition (the 0,0 coordinate) by showing the distribution of simulated ICERs across four quadrants: 1) the Northeast (NE) quadrant, which means that the intervention is more effective and more costly than CAU, 2) the Southeast (SE) quadrant, indicating that the intervention is more effective and less costly, 3) the Southwest (SW) quadrant, indicating that the intervention is less effective and less costly and 4) the Northwest (NW) quadrant, indicating that the intervention is less effective and more costly. An ICER in the SE and NW quadrant is negative, which represents the situation in which the intervention is either clearly dominant over (SE) or inferior to (NW) CAU. An ICER in the SW or NE quadrant is positive, which means, from a cost-effectiveness perspective, that the intervention is more favorable than the control condition only when the ICER is lower than the maximum willingness to pay (WTP max) per unit effect. The WTP max is the maximum expense a society is willing to pay for better outcomes (parenting quality, in this study). Since no acknowledged threshold, i.e. WTP max, is available for the HOME outcome measure, a CEAC was created for each perspective (Fig. 2d,e,f). The CEAC shows the likelihood of PBCM being favorable over the control intervention for several different hypothetical maximum WTPs.	

#### Sensitivity analysis

**Fig. 1:**
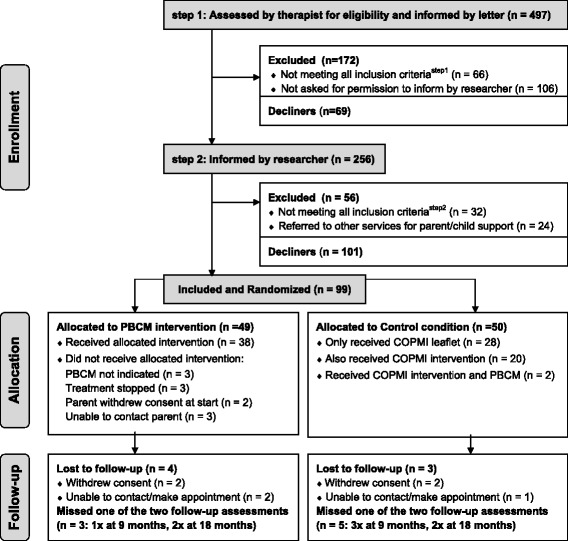
Flow chart of the participating families through recruitment and the study. Information about excluded patients and decliners: In step 1, 106 families were not contacted by the researcher due to lack of continuity or ending of contact between therapist and patient, or not being able to contact them in person by phone. In step 2, 32 families were found to be ineligible because the children were not in the required age category or because the child had been diagnosed with mental health problems; 24 families were referred by the researchers to relevant parental support services or child services; and 101 families declined to participate, mostly because they were not interested in support or in participating in a research project

For each perspective, several additional sensitivity analyses were performed to test the robustness of the ICERs calculated in the base case scenario. First, to examine the impact of cost outliers (i.e. high cost families) on the calculated cost-effectiveness, ICERs were calculated based on data in which the top 5 % cost outliers were excluded (alternative scenario A). Second, to assess the impact of imputation, the same analyses were conducted on complete cases (alternative scenario B). Third, to examine the effects of implementing the intervention, a subgroup analysis (alternative scenario C) was carried out on the sample that actually received PBCM (*N* = 38) (see flow chart, Fig. 1). Finally, apart from the routine unadjusted base case scenario, CEAs should include an alternative scenario in which baseline cost differences are adjusted [43]. To adjust for baseline cost differences between the two conditions in this study, ICERs were calculated based on mean difference adjustments (alternative scenario D). By using this method, the mean difference in costs between conditions at baseline is first extrapolated to equal the length of the follow-up period (i.e. 18 months), and subsequently subtracted from the total post-randomization costs (intervention costs and costs for services after randomization) of the condition with the highest baseline costs [43]. The base case scenario and alternative scenarios resulted in a total of fifteen ICERs. Finally, we compared reported contacts with registered community mental health service contacts to estimate the reliability of self-reporting.

## Results

### Participant flow

As can be seen in the flow chart (Fig. 1), families were recruited in two steps. In the first step, researchers screened each therapist’s caseload for eligible families, using the exclusion criteria. This resulted in 497 patients, who were approached by letter, in which the therapists asked the patients for permission to be contacted by the researchers. In the second step, the researchers contacted 256 eligible and interested families, checked whether the parent(s) were interested in PBCM, and checked all inclusion- and exclusion criteria. Ninety-nine families were included and randomly allocated to either PBCM (*n* = 49) or to the control condition (*n* = 50). Of the 49 families allocated to PBCM, 38 (77 %) actually did receive the intervention. The reasons for not receiving PBCM were: PBCM was not indicated according to the PBCM coordinator, treatment was terminated, the parents withdrew consent at the start, or the PBCM coordinator was not able to contact parents. Of the 50 families in the control group, 22 (44 %) made use of the COPMI team for consultation or of COPMI groups, and two were also referred to the PBCM intervention. Dropout was low in both arms (Fig. 1), namely four of the 49 families in the PBCM group and three of the 50 in the control group (χ2 = .18, df = 1, *p* = 0.68), and these were not related to characteristics or outcome measures. At baseline, 99 files were available, 86 files were available at the second assessment, and 88 files at the third assessment. A total of 82 families (83 %) had complete datasets for the HOME.

### Baseline data

As shown in Table 2, in most families the mother was the primary patient, and most parents were diagnosed with depressive or anxiety disorders. Half of the families included were single parents, and two-thirds were of ethnic minorities. The mean number of children was 2.1, and most children were of primary school age. The mean T-score on HOME was 50 (an average score compared with the population norm in the manual [28]), and the mean number of risk factors was five on a scale of sixteen. The PBCM group contained significantly more single parent families, more families from ethnic minorities, and the mean age of the index child was significantly higher than in the control group. The groups did not differ on other aspects.

**Table 2:** Baseline characteristics and baseline scores of families in the experimental group and in the control group

Variable	Experimental Group (*n* = 49)				Control Group (*n* =50)				Difference (df)	*P*
Primary patient and family structure									χ^2^ = 4.45 (1)	0.035^ab^*
Mother/single, *N* (%)	28	(57 %)			18	(36 %)				
Mother/two-parent family, *N* (%)	15	(31 %)			26	(52 %)				
Father/two-parent family, *N* (%)	2	(4 %)			2	(4 %)				
Mother and father, *N* (%)	4	(8 %)			4	(8 %)				
Diagnosis	Mothers		Fathers		Mothers		Fathers		χ^2^ = 0.98 (2)	0.976^b c^
Depressive and anxiety disorders, *N* (%)	36	(77 %)	4	(67 %)	36	(75 %)	4	(67 %)		
Other Axis I disorders, *N* (%)	8	(17 %)	2	(33 %)	9	(19 %)	2	(33 %)		
Personality disorders, *N* (%)	3	(7 %)	-	-	3	(6 %)	-	-		
Comorbidity, severity and chronicity										
Comorbidity, *N* (%)	20	(43 %)	3	(50 %)	25	(52 %)	2	(33 %)	χ^2^ = 0.87 (1)	0.352^b^
Severity of illness CGI, mean (sd)	4.53	(1.10)	4.38	(0.50)	4.51	(0.92)	4.00	(1.0)	t = 0.79 (93)	0.917^b^
Chronic course of illness > 2 years, *N* (%)	18	(38 %)	2	(33 %)	24	(48 %)	2	(33 %)	χ^2^ = 0.68 (1)	0.257^b^
Ethnicity									χ^2^ = 7.30 (1)	0.007*
Ethnic minority, *N* (%)	39	(80 %)			27	(54 %)				
Morocco, *N* (%)	11	(22 %)			8	(16 %)				
Turkey, *N* (%)	9	(18 %)			6	(12 %)				
Surinam, *N* (%)	8	(16 %)			6	(12 %)				
Netherlands Antilles, *N* (%)	5	(10 %)			2	(4 %)				
Other country, *N* (%)	6	(12 %)			5	(10 %)				
Children										
Number, mean (sd)	2.10	(0.98)			2.16	(1.02)			t = -0.29 (97)	0.774
Children 0-3 years (*N*)	27	(82 %)			35	(90 %)			χ^2^ = 2.01 (3)	0.570
Children 4-12 years (*N*)	61	(24 %)			63	(34 %)			χ^2^ = 2.24 (3)	0.524
Children 13-20 years (*N*)	13	(18 %)			9	(14 %)			χ^2^ = 0.77 (2)	0.682
Male gender index child, *N* (%)	25	(51 %)			30	(60 %)			χ^2^ = 0.81 (1)	0.619
Age index child, mean (sd)	6.53	(2.19)			5.64	(1.76)			t = 2.25 (97)	0.027*
HOME total score at baseline, mean (sd)	48.59	(10.79)			51.38	(9.05)			t = -1.40 (97)	0.166
Costs at baseline										
Healthcare costs (Euros, 2012)	5.156				6.275					
Childcare costs (Euros, 2012)	2.687				3.751					
Inter-sectoral costs (Euros, 2012)	1.411				1.009					
Other										
Number of risk factors, mean (sd)	5.20	(1.38)			5.02	(1.48)			t = 0.64 (97)	0.524
Receiving social benefits, *N* (%)	23	(47 %)			15	(30 %)			χ^2^ = 3.00 (1)	0.083

* *p* < 0.05

^a^tested for single versus two parents

^b^There were 47 mothers and 6 fathers in the experimental group; there were 48 mothers and 6 fathers in the control group. This is the reason that the sum of the figures in the first three rows is not 49 and 50.

^c^tested for mothers and not for fathers, as both groups had only 6

### Costs

The mean intervention costs for PBCM (*n* = 49) were 1,685 Euros, and mean costs for the control condition (*n* = 50) were 229 Euros (Table 3). Intervention costs for the subgroup of allocated families who did receive the intervention were 2,053 Euros in the PBCM group (*n* = 38) and 285 Euros for the control group (*n* = 22) (data not shown). Therefore, depending on the approach, the intervention costs of PBCM are 1,456 (*n* = 49) or 1,768 Euros (*n* = 38) more costly in comparison with CAU.

**Table 3:** Mean per-family costs by condition and measurement (in Euros, indexed for 2012)

		Follow-up T0-T1, (first 9 months)		Follow-up T1-T2, (10 to 18 months)		Total T0-T2, (full 18 months)	
		PBCM	Control	PBCM	Control	PBCM	Control
Intervention Costs						€ 1,685	€ 229
Service Costs	Healthcare costs	€ 5,875	€ 6,528	€ 5,452	€ 4,462	€ 11,327	€ 10,990
	Mental healthcare	€ 2,650	€ 1,963	€ 1,861	€ 1,340	€ 4,511	€ 3,303
	Primary care (other)	€ 525	€ 715	€ 734	€ 391	€ 1,259	€ 1,106
	Secondary care (other)	€ 1,044	€ 1,820	€ 1,233	€ 857	€ 2,277	€ 2,677
	Preventive family support	€ 1,399	€ 1,908	€ 1,350	€ 1,651	€ 2,749	€ 3,559
	Specialized child services	€ 257	€ 122	€ 274	€ 223	€ 531	€ 345
	Total healthcare perspective					€ 13,012	€ 11,219
	Childcare costs	€ 2,304	€ 3,010	€ 2,401	€ 2,750	€ 4,705	€ 5,760
	Informal childcare	€ 1,115	€ 1,341	€ 1,169	€ 1,286	€ 2,284	€ 2,627
	Professional childcare	€ 1,189	€ 1,669	€ 1,232	€ 1,464	€ 2,421	€ 3,133
	Total social care perspective					€ 17,717	€ 16,979
	Costs outside care sector	€ 1,156	€ 522	€ 930	€ 1,708	€ 2,086	€ 2,230
	Educational sector	€ 685	€ 107	€ 553	€ 1,302	€ 1,238	€ 1,409
	Criminal justice sector	€ 238	€ 38	€ 52	€ 169	€ 290	€ 207
	Debt restructuring	€ 233	€ 377	€ 325	€ 237	€ 558	€ 614
	Total societal perspective					€ 19,805	€ 19,209

During the whole follow-up period of eighteen months, the mean healthcare costs per family in the PBCM condition were 11,327 Euros, which was higher than in the control condition (10,990 Euros). Childcare costs were lower in the PBCM condition, namely 4,705 Euros versus 5,760 Euros in the control condition. The same goes for costs in other sectors, where mean costs in the PBCM condition were 2,086 Euros and mean costs in the control condition were 2,230 Euros. Table 3 also provides the mean per-family costs from each perspective (intervention costs plus costs for use of services), which were used for calculating the ICERs. Differences in costs between T1 and T2, such as differences in costs in the educational sector, can be explained by irregular use of services.

### Incremental costs

Table 4 (upper panel) shows costs per condition for the base case scenario. The difference in average per-family costs between the PBCM and control condition varies for each of the three perspectives, namely 1,793 Euros from the healthcare perspective, 738 Euros from the social care perspective and 596 Euros from the societal perspective. For each perspective, costs were higher in the PBCM condition.

**Table 4:** Summary statistics of the base case analyses and sensitivity analyses from three perspectives

Perspective^a^	Condition	Costs, €^b^	Effect^c^	ICER^d^	Northeast	Northwest (inferior)	Southwest	Southeast (dominant)
Base case scenario								
*(imputed data, including cost outliers* ^e^ *)*								
Healthcare	Control (*n = 50*)	11,219	-1.89					
	PBCM (*n = 49*)	13,012	1.93	461	78 %	2 %	1 %	20 %
Social care	Control (*n = 50*)	16,979	-1.89					
	PBCM (*n = 49*)	17,717	1.93	215	60 %	1 %	1 %	37 %
Societal	Control (*n = 50*)	19,209	-1.89					
	PBCM (*n = 49*)	19,805	1.93	175	59 %	1 %	1 %	39 %
Alternative scenario A								
*(imputed data, excluding cost outliers)*								
Healthcare	Control (*n = 47*)	8,969	-1.28					
	PBCM (*n = 47*)	11,564	1.70	776	90 %	6 %	0 %	4 %
Social care	Control (*n = 47*)	14,422	-1.40					
	PBCM (*n = 47*)	16,138	1.70	517	81 %	4 %	1 %	15 %
Societal	Control (*n = 47*)	16,634	-1.82					
	PBCM (*n = 47*)	18,194	1.70	410	76 %	3 %	1 %	21 %
Alternative scenario B								
*(complete cases, including cost outliers)*								
Healthcare	Control (*n = 41*)	11,475	-2.06					
	PBCM (*n = 41*)	13,480	2.34	446	79 %	1 %	0 %	20 %
Social care	Control (*n = 41*)	17,765	-2.06					
	PBCM (*n = 41*)	18,375	2.34	133	58 %	1 %	1 %	40 %
Societal	Control (*n = 41*)	20,242	-2.06					
	PBCM (*n = 41*)	19,621	2.34	dominant^f^	41 %	0 %	1 %	58 %
Alternative scenario C								
*(imputed data, including cost outliers, PBCM-families who received the intervention)*								
Healthcare	Control (*n = 48*)	10,933	-1.65					
	PBCM (*n = 38*)	14,579	2.24	897	93 %	2 %	0 %	5 %
Social care	Control (*n = 48*)	16,140	-1.65					
	PBCM (*n = 38*)	19,522	2.24	843	90 %	2 %	0 %	8 %
Societal	Control (*n = 48*)	18,458	-1.65					
	PBCM (*n = 38*)	20,736	2.24	558	79 %	2 %	0 %	20 %
Alternative scenario D								
*(imputed data, including cost outliers, mean difference adjustment)*								
Healthcare	Control (*n = 50*)	8,981	-1.89					
	PBCM (*n = 49*)	13,012	1.93	1,031	95 %	2 %	0 %	3 %
Social care	Control (*n = 50*)	12,613	-1.89					
	PBCM (*n = 49*)	17,717	1.93	1,313	96 %	2 %	0 %	2 %
Societal	Control (*n = 50*)	15,647	-1.89					
	PBCM (*n = 49*)	19,804	1.93	1,059	92 %	2 %	0 %	6 %

^a^In the analyses either 1) intervention and healthcare costs (healthcare perspective), 2) intervention, healthcare and child care costs (social care perspective) or 3) all measured costs (societal perspective) were included

^b^Costs per family at 2012 prices

^c^Average effectiveness (T-score) compared with the baseline assessment

^d^The presented median ICER is the 50th percentile of 5000 bootstrap replications of the ICER

^e^Differences in effects between the three perspectives are caused by the exclusion of cost outliers, which differed among the three perspectives

^f^ Lower incremental costs and a positive incremental effect of PBCM in comparison with the control condition leads to a negative ICER, which means that PBCM is superior to the control condition on cost-effectiveness

### Incremental effects

Table 4 (upper panel) shows the effects per condition for the base case scenario. PBCM had a positive effect on parenting quality, with an increase of the HOME T-score of 1.93 from 48.59 (SD 10.79) at baseline to 50.52 (SD 11.92) after eighteen months. In the control condition the HOME T-score decreased by 1.89 points, from 51.38 (SD 9.05) to 49.49 (SD 6.48). The mean incremental effect per family between the PBCM and control condition was, therefore, 3.82, and did not change with perspective, since the change of perspective within the base case scenario stipulated only a change in costs.

### Incremental cost-effectiveness

From all three perspectives, costs per unit of the outcome measure (HOME T-score) were higher for the PBCM condition in comparison with the control condition. Since PBCM was more effective than CAU, this resulted in positive ICERs (Table 4, upper panel). However, ICERs differ for each perspective, varying from 461 Euros (healthcare perspective) to 215 Euros (social care perspective) to 175 Euros (societal perspective) per one point improvement on the HOME T-score. Differences can be explained by healthcare costs being higher and childcare costs and costs in other sectors being lower for the PBCM condition in comparison with the control condition (Table 3).

**Fig. 2:**
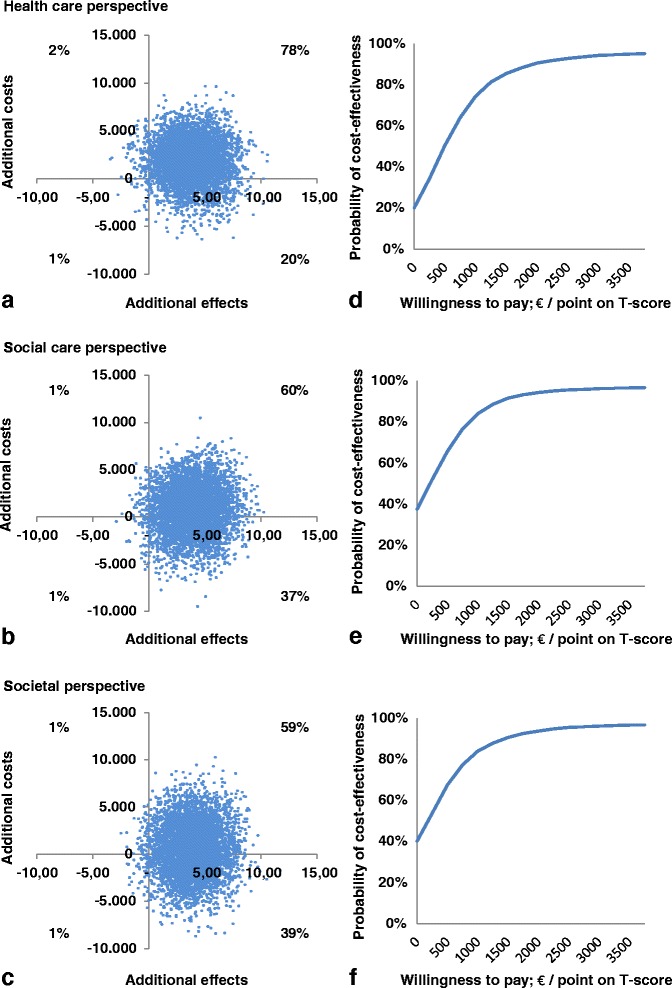
Cost-effectiveness planes and CEACs from the three perspectives. Scatterplots of simulated incremental cost-effectiveness ratios (*n* = 5000) on cost-effectiveness planes (**a, b, c**) and CEACs (**d, e, f**) for the PBCM versus the control condition from the healthcare perspective (**a, d**), social care perspective (**b, e**) and societal perspective (**c, f**)

The cost-effectiveness planes (Fig. 2a,b,c) show differences in distributions of the 5,000 simulated ICERs across the four quadrants between the CEAs carried out from the three perspectives. Corresponding with median ICERs presented in Table 4, the majority of simulated ICERs are located in the NE quadrant. However, the distribution of the simulated ICERs among the two eastern quadrants differs among the perspectives. Notable is the shift of the cloud of ICERs towards the SE quadrant in the analysis carried out from the societal perspective (39 %) and the social care perspective (37 %) in comparison with the analysis carried out from the healthcare perspective (20 %).

The percentages mentioned above equal the probabilities of PBCM being cost-effective at a WTP max of 0 Euros – i.e. the situation in which one is not willing to pay for this intervention - in the CEACs (Fig. 2d,e,f), and explain why for low WTP thresholds the probability of PBCM being cost-effective over the control intervention is lower from a healthcare perspective than it is from broader perspectives. However, since for all three perspectives the vast majority of simulated incremental effects are in the NE, all CEACs rise when the WTP max increases and all asymptote close to 100 % around 2,500 Euros. The probabilities of PBCM being cost-effective do not differ among perspectives for WTP thresholds higher than 2,500 Euros (Fig. 2d,e,f).

### Sensitivity analysis

The results of the sensitivity analyses are presented in the second to fifth panel of Table 4. In scenario A (second panel), ICERs were higher than in the base case scenario. This can be explained by the fact that in all three perspectives, the majority of cost outliers - three or four out of the five excluded - were families in the control condition. In scenario B (third panel), in which incomplete cases were removed before data was analyzed, ICERs were lower than in the base case scenario. The analysis conducted from a societal perspective resulted in an ICER of -143, with 58 % of the cloud situated in the SE quadrant, and was therefore marked as ‘dominant’ in Table 4. Scenario C – i.e. the subgroup analyses (fourth panel) - resulted in ICERs higher than in the base case scenario. In all these scenarios, ICERs were highest from the healthcare perspective and lowest from the societal perspective. In scenario D (fifth panel), in which the analyses were performed based on mean baseline difference adjustments, the ICERs were highest in all scenarios, varying from 1,031 Euros (healthcare perspective) to 1,313 Euros (social care perspective) to 1,059 Euros (societal perspective). This can be explained by the higher baseline costs in the control condition for all three perspectives. Cost-effectiveness planes and CEACs of the sensitivity analyses can be obtained via supplementary material which is published online (Additional file 2). To estimate the reliability of self-reporting, we compared reported contacts with registered community mental health service contacts. These showed a significant underreporting of 1,543 Euros in the follow-up period (t = 4.06, df = 87, *p* = 0.000). No differences in underreporting were found between the intervention and control condition (t = 1.09, df = 86, *p* = 0.278). No correction for underreporting was made in the analyses of costs and ICERs.

## Discussion

### Main findings

The aim of this study was to (a) examine the costs and cost-effectiveness of PBCM and (b) answer the question whether shifting from a narrow (healthcare) perspective to broader perspectives, in which either childcare costs (social care perspective) or childcare costs and other ICBs (societal perspective) were included, results in a change in the cost-effectiveness of PBCM.

Comparing the total costs (intervention costs plus costs of service utilization) in the PBCM group and the control group, the conclusion is that PBCM is more costly. The extra costs of PBMC ranged from 1,793 Euros from a healthcare perspective to 738 Euros from a social care perspective to 596 Euros from a societal perspective. The savings in the last two perspectives can be attributed to lower costs for childcare, debt reconstruction and in the educational sector of the PBCM group in comparison with the control group.

PBCM had better effects on parenting quality than CAU, but also had higher costs. Therefore, ICERs were positive. The cost differences among perspectives are reflected in the ICERs; the ICER is highest in the analysis conducted from the narrowest perspective (healthcare, 461 Euros), lower in the analysis conducted from a broader perspective (social, 215 Euros), and lowest in the analysis conducted from the broadest perspective (societal, 175 Euros). Sensitivity analyses based on excluding cost outliers, excluding incomplete cases and the subgroup analysis, confirmed that a broader perspective leads to a lower ICER. It can be concluded that, for this study, the choice of perspective has had an impact on the outcome of the analysis. However, the difference between ICERs is larger between the healthcare perspective and the social care perspective (246 Euros) than it is between the social care perspective and the societal perspective (40 Euros). This shows that the impact of including ICBs other than childcare on the outcomes of this CEA was fairly limited. Nevertheless, they did show an impact on the results.

Whether PBCM is considered cost-effective over CAU depends on the WTP max per point gain on the HOME T-score (Fig. 2d,e,f). The probabilities of PBCM being cost-effective start at 20 % (healthcare perspective), 37 % (social care perspective) and 39 % (societal perspective) at a WTP max of 0 Euro and increase with an increasing WTP max. For thresholds lower than 2,500 Euros, the chances of PBCM being favorable over the control intervention are higher when a broader perspective is adopted. For thresholds higher than 2,500 Euros, there is a near 100 % probability of PBCM being cost-effective regardless of the perspective chosen.

### Strengths and limitations

This study was the first to assess the costs of a preventive family intervention for COPMI families and relate it to parenting outcomes. The strengths of this study are the randomized controlled design and the broad range of sensitivity analyses conducted to test the robustness of the analysis in the base case scenario. The sensitivity analyses were limited to costs and not to effects; the analyses showed no outliers on effects and showed no significant baseline differences in the HOME T-scores. Furthermore, the real world setting strengthens the generalizability of the results. The PBCM method and the population in this study represent the state of the art.

The study has several limitations, which should be addressed for the interpretation of the findings. First, no adequate instruments were available to assess the quality adjusted life years (QALYs) of young COPMI. However, the HOME instrument is a valid instrument, used widely and internationally to measure parenting quality, and it can be interpreted as a proxy for quality of life; the HOME measures many aspects of parenting and the home environment which are suggested as being essential within the concept of quality of life for COPMI’s physical, emotional, social and material well-being [22]. Nevertheless, it should be noted that the HOME has ceiling effects [31], which may have reduced sensitivity for effects and for PBCM’s cost-effectiveness.

Second, although the HOME T-score was a clinically relevant outcome measure for parenting quality, its use within a CEA is new. The lack of clinical cut-off scores impedes interpretation of improvement in parenting quality, in terms of the economic value, for policy making. Also, no thresholds for WTP on costs per unit effect are available for the HOME T-score, as are widely used outcome measures capturing utility such as the QALY [45, 46]. Since the intervention is both more costly and more effective than CAU, the lack of WTP thresholds makes it hard to interpret the economic value of the improvement of parenting quality. However, the CEACs provide decision supportive information because these provide cost-effectiveness probabilities for a wide range of hypothetical thresholds for all analyses. Furthermore, looking at effects, prospective studies on the long-term outcomes of parenting quality (measured by the HOME) showed positive health or societal outcomes. These studies showed low to moderate correlations with (later) child development such as intelligence, academic achievement, school performance, language development, social competence, classroom behavior, peer acceptance, and emotional health [47]. Furthermore, HOME scores were shown to be related to such health issues as malnutrition, failure-to-thrive, and child abuse [48].

Third, given limitations regarding the feasibility of assessing the costs for vulnerable parents within an RCT, we chose to focus on services which are important partners for PBCM, such as youth care, childcare, education, and the justice and social systems. Productivity costs in parents were not measured. Including this ICB within the analysis conducted from a societal perspective might have had an influence on the cost-effectiveness. Also, self-reported service utilization may have distorted the calculation of costs. As no differences in underreporting were found between the intervention and control condition, the effect of self-reporting on the cost-effectiveness is not obvious. Furthermore, we did not closely monitor the occurrence of waiting lists for the families during the study, though none was reported in the PBCM files. But waiting lists might have obscured the results of this study.

Fourth, differences in the baseline costs of both groups substantially affected ICERs. After adjusting for differences in baseline costs, ICERs climbed to more than 1,000 Euros. The differences in costs are probably related to differences in family composition, such as being a one-parent family, and the age of the children. The needs and barriers for different kind of services might vary depending on the family composition. For instance, savings in childcare might also be related to differences in family composition, since the control group contained more young preschool children (35 versus 27). However, it is hard to predict how this affects the total costs. We found no relation between baseline total costs and one/two-parent families, the number of children under the age of four or ethnicity (data not shown). Still, incorporating family characteristics (such as composition, ages of family members) in CEAs remains a challenge, especially in multi-ethnic samples.

Fifth, the study was conducted on a relatively small and rather heterogeneous sample (e.g. parental diagnosis, family composition, ethnicity, and source of income). The effect of scores of single families on variances in effects and costs, such as outliers, might have affected the cost-effectiveness found in this study. This is reflected in the differences between the ICERs in the base case scenario and alternative scenario A, where ICERs were calculated excluding cost outliers.

Finally, the chosen time frame of eighteen months might not have been long enough to study all meaningful effects and costs, such as long-term ICBs related to the school career, work or criminality of youngsters. Moreover, the young age of the children and absence of evident behavioral problems may have reduced the chance of finding these ICBs. The need for a long time frame for cost-effectiveness studies on preventive family support has been shown in the Nurse-Family Partnership study [49]. Long-term prospective studies are needed to explore the effects in children and co-occurring costs in the long run. As a consequence of the limitations described above, it is difficult to determine whether PBCM provides “value for money”. Nevertheless, in this study PBCM showed better effects on parenting quality than CAU and this study gives an overall estimate of the additional costs.

## Conclusion

This study is the first economic evaluation of a family-focused preventive COPMI approach in psychiatric and family services. The results of this study show, from both a healthcare and a societal perspective, that the intervention is both more costly and more effective than CAU. Since no WTP study was conducted, no conclusive ‘yes’ or ‘no’ can be provided to the question whether the intervention is cost-effective. However, as mentioned earlier, the CEACs provide decision supportive information. Furthermore, the found size of the effect and savings in several sectors support focusing on prevention and on the health of vulnerable children and families in all policies.

The results of our study may be of interest for community policy makers and stakeholders in health policy and youth care when optimizing service systems for COPMI families within a framework of restricted financial resources. It underscores the importance of evaluating costs and benefits in other sectors when planning and evaluating innovative integrative services for children or families at risk. However, before implementing PBCM on a wider scale, replication studies, preferably along with cost-utility analyses measuring costs, benefits and QALYs of young COPMI, and multi-center studies of case management programs for COPMI families are needed. These studies could also help to gain insight over the various effects and the economic costs and benefits in subgroups, to better indicate which families are best served. Studies in systems with lower provision of and/or accessibility to services in different countries are needed, since the current Dutch service system is one of the richest and egalitarian ones in the world, with good accessibility for poor families. This study punctuates the importance of choosing a broad societal perspective in economic evaluations. ICBs should be and already are increasingly considered in underpinning (the financing of) health policies.

## Abbreviations

CAU, care as usual; CEA, cost-effectiveness analyses; CEAC, cost-effectiveness acceptability curve; COPMI, Children of Parents with a Mental Illness; HOME, Home Observation for Measurement of the Environment; ICBs, inter-sectoral costs and benefits; ICER, incremental cost-effectiveness ratio; PBCM, preventive basic care management; QALY, quality adjusted life year; VHO, Vragenlijst Hulp en Ondersteuning (family support questionnaire); WTP, willingness to pay

## Additional files

Additional file 1: Services and sources for prices. This categorization of services in Health Care, Youth Care, Childcare and services in other sectors follows the current system in the Netherlands. Health Care and Youth Care are services financed by health insurance and the government for the prevention and treatment of somatic, mental, and developmental problems. Childcare is financed by parents themselves for babysitting or kindergarten. Other sectors include the educational sector and criminal justice sector. For each service sources are given for pricing. (DOCX 23 kb) Additional file 2: Cost-effectiveness planes and CEACs for alternative scenarios. This figure shows the scatterplots of simulated incremental cost-effectiveness ratios (*n* = 5000) on cost-effectiveness planes and CEACs for the PBCM versus the control condition in four alternative scenarios: 1) excluding outliers (alternative scenario A), 2) based on complete cases (alternative scenario B), 3) the sample that actually received the intervention (alternative scenario C) and 4) corrected for baseline cost differences (alternative scenario D). (PDF 3713 kb)
