# Supplementary material for: Cost-effectiveness of preventive case management for parents with a mental illness: a randomized controlled trial from three economic perspectives
Source: BMC Health Serv Res. 2016 Jul 7;16:228. doi: 10.1186/s12913-016-1498-z (PMC4937554; doi:10.1186/s12913-016-1498-z)
Supplement: Additional file 1: — Services and sources for prices. This categorization of services in Health Care, Youth Care, Childcare and services in other sectors follows the current system in the Netherlands. Health Care and Youth Care are services financed by health insurance and the government for the prevention and treatment of somatic, mental, and developmental problems. Childcare is financed by parents themselves for babysitting or kindergarten. Other sectors include the educational sector and criminal justice sector. For each service sources are given for pricing. (DOCX 23 kb) [file 12913_2016_1498_MOESM1_ESM.docx]

**Appendix 1 Services and sources for prices**

This categorization of services in Health Care, Youth Care, Child Care and services in other sectors follows the current system in the Netherlands. Health Care and Youth Care are services financed by health insurances and the government for prevention and treatment of somatic, mental, and developmental problems. Child Care is financed by parents themselves for babysitting or kindergarten. Other sectors include the school en criminal system. For each service sources are given for pricing.

| *Category* | *Included services* | *Source* | *Reference, or Alternative Method for pricing (AM)* |
| --- | --- | --- | --- |
| *Health Care* | | | |
| Mental health care | primary mental health care, community mental health services, psychiatric clinics | (1) | (1) Hakkaart-van Roijen L, Tan SS, Bouwmans CA. *Manual for cost studies. Methods and reference prices for economic evaluations in health care.* *Updated version. [Handleiding voor kostenonderzoek. Methoden en referentieprijzen voor economische evaluaties in de gezondheidszorg.] Geactualiseerde versie 2010].* Diemen: College voor Zorgverzekeringen, 2011. |
| Other Primary  health care | GP, paramedical services, logopedics, dietician, health and safety service,  social welfare work | (1) |  |
|  | alternative medicine | (2) | (2) Bouwmans CAM, Schawo SJ, Jansen DEMC, Vermeulen KM, Reijneveld SA, Hakkaart-van Roijen L. *iMTA Questionnaire Intensive Youth Care [Handleiding*  *Vragenlijst Intensieve Jeugdzorg Zorggebruik en productieverlies]*. 2012; Available from: https://[www.bmg.eur.nl/fileadmin/ASSETS/bmg/Onderzoek/Onderzoeksrapporten___Working_Papers/2012.06_-_Handleiding_Vragenlijst_Intensieve_Jeugdzorg.pdf](http://www.bmg.eur.nl/fileadmin/ASSETS/bmg/Onderzoek/Onderzoeksrapporten___Working_Papers/2012.06_-_Handleiding_Vragenlijst_Intensieve_Jeugdzorg.pdf). |
| Other Secondary  health care | somatic (general/academic) hospitals, emergency room, revalidations centers | (1) |  |
|  | specialized clinics for obesity  specialized burns department | AM | Costs were estimated to be equivalent to costs of general hospital admissions, of which prices were given in (1). |
| *Youth Care* Preventive Family Support Services | Youth and Family Centers | AM | Costs were estimated to be equivalent to costs of personal nursing of which prices were given in (1). |
|  | domestic services | (1) |  |
|  | preventive home-based family care | (3) | (3) Nederlands Jeugd instituut. *Prices standard research Youth and parenting support Noord-Brabant.* *[Normprijzenonderzoek Jeugd & Opvoedhulp Noord-Brabant.]* Utrecht: NJi, 2010. http://www.nji.nl/nl/Normprijzen_jeugd _opvoedhulp_Brabant.pdf |
|  | preventive orthopedagogical services (Salvation Army, Preventive Youth Care) | AM | Costs were estimated based on equivalent services for social work, of which cost prices were available in (1). |
|  | parenting classes and parenting education | AM (4) | Costs were estimated based on equivalent to domestic services, of which prices were given in (4) Drost RMWA, Paulus ATG, Ruwaard D, Evers SMAA. *Manual intersectoral costs and benefits of (preventive) interventions: Classification, identification and prices.* [*Handleiding intersectorale kosten en baten van (preventieve) interventies: Classificatie, identificatie en kostprijzen.]* Maastricht: Maastricht University, Department of Health Services Research, 2014. |
|  | preventive mental health education for children and parents | AM | Price as billed by the institute [www.context.nl](http://www.context.nl) |
|  | family coaches of Youth and Family Centers | AM | Costs were estimated based on equivalent services for social work, of which cost prices were given in (1). |
|  | home-based family support by non-professionals | AM | Costs were estimated based on equivalent services for domestic services, of which cost prices were given in (4). |
| *Youth Care* Specialized Youth Care Services | youth care services  (semi-residential care, residential care), | (3) |  |
|  | foster care and secure care | (5) | (5) [https://www.pleegzorg.nl/media/uploads/ nieuws/indexering_ pleegvergoeding_2012_staatscourant_stcrt-2011-23215.pdf](https://www.pleegzorg.nl/media/uploads/%20nieuws/indexering_%20pleegvergoeding_2012_staatscourant_stcrt-2011-23215.pdf) |
|  | child protection and probation services | AM | Costs were estimated to be equivalent to costs of social work, of which prices were given in (1). |
|  | Youth Care Agencies (indication assessment) | (1) |  |
|  | intensive ambulatory home based specialized support for multi-problem families | (3) |  |
|  | care for youth with mental and/or cognitive disabilities | (6) | (6) NZA (2012). BELEIDSREGEL CA-300-487, Prestatiebeschrijvingen en tarieven extramurale zorg 2012, Kenmerk CA- 300-487, Bijlage 14 bij circulaire AWBZ/Care/11/9c. |
|  | | | |
| *Child Care* | | | |
| Informal Child Care | child care given by nonprofessionals (babysitter, granny) | (2) |  |
| Professional Child Care | child care services, such as kindergarten | (2) |  |
|  | | | |
| *Other Sectors* | | | |
| Educational sector | school attendance officer, interne special education teacher, special education, specialized educational services | (4) |  |
| Criminal/justice sector | lawyers, police, court | (4) |  |
| Debts restructuring services | debts restructuring services | AM | Costs were estimated to be equivalent to the costs of social work, of which prices were given in (1). |
