# Supplementary material for: Cost-effectiveness of preventive case management for parents with a mental illness: a randomized controlled trial from three economic perspectives
Source: BMC Health Serv Res. 2016 Jul 7;16:228. doi: 10.1186/s12913-016-1498-z (PMC4937554; doi:10.1186/s12913-016-1498-z)

Alternative scenario A

Healthcare perspective

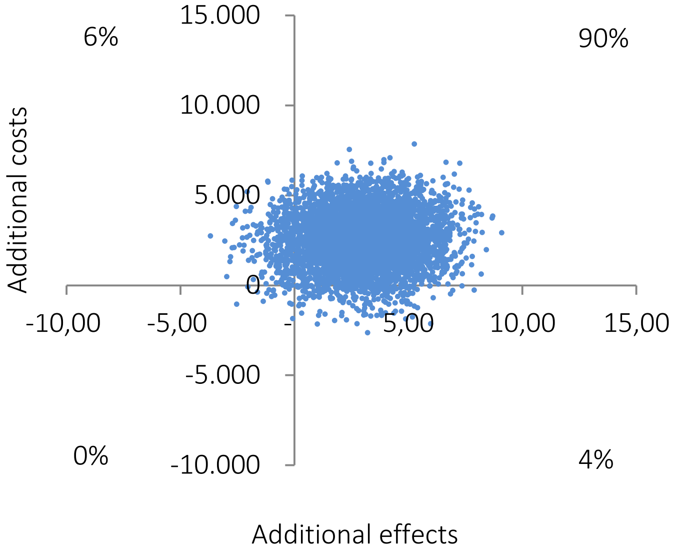

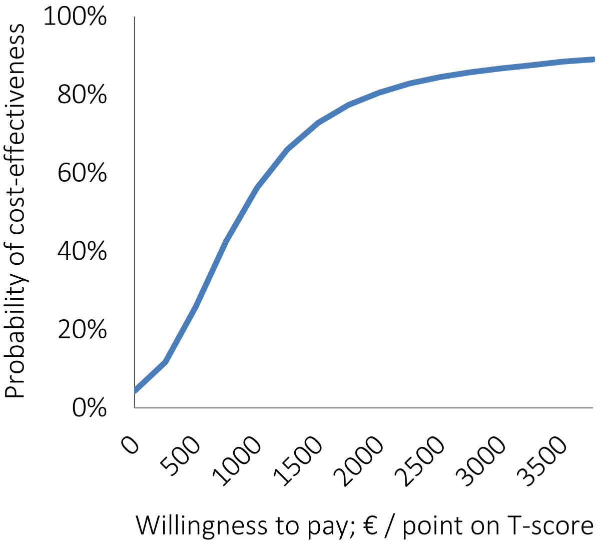

Social care perspective

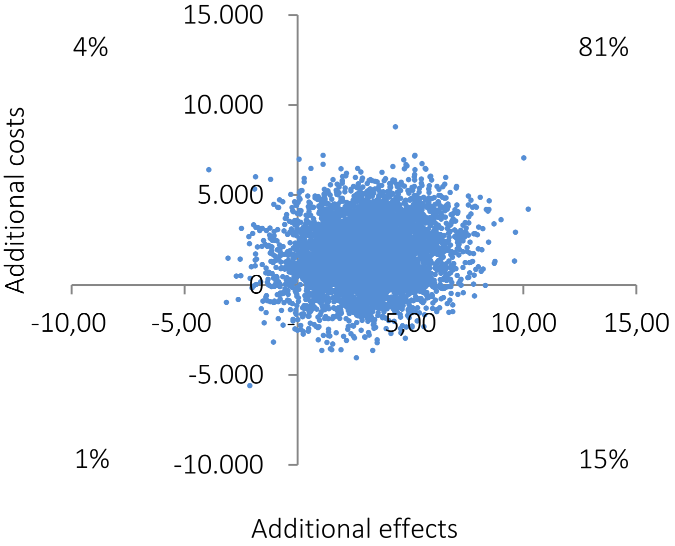

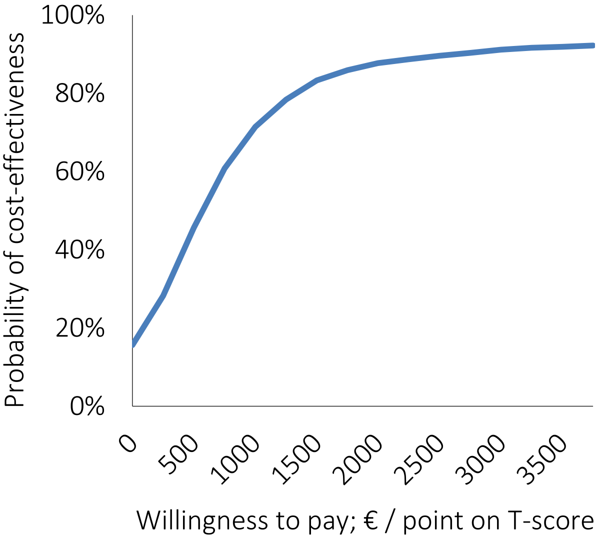

Societal perspective

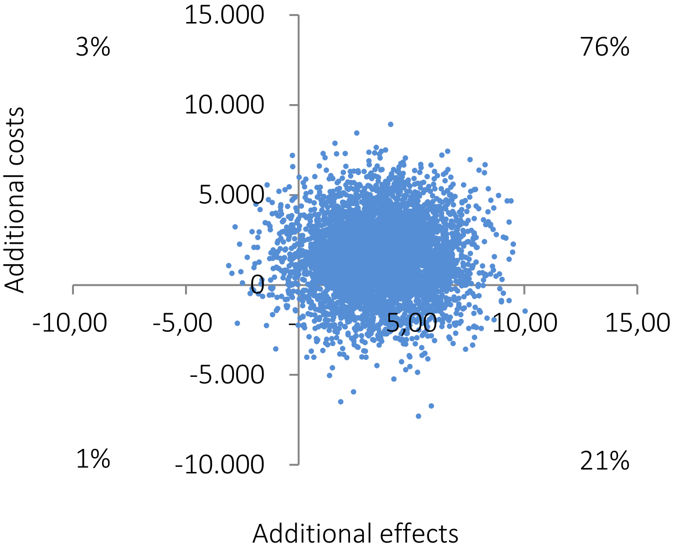

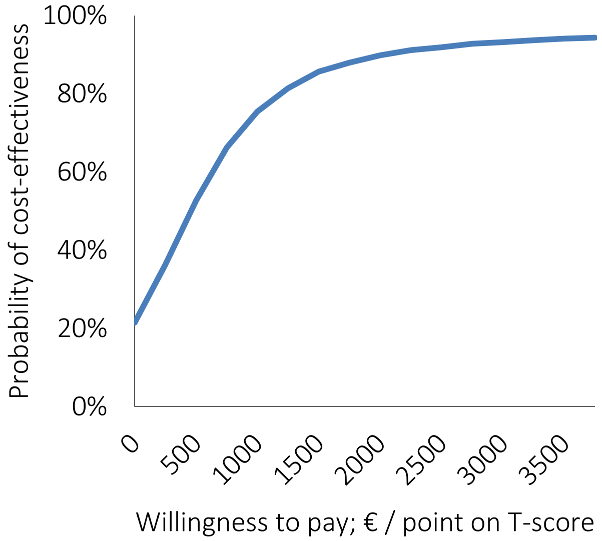

Alternative scenario B

Healthcare perspective

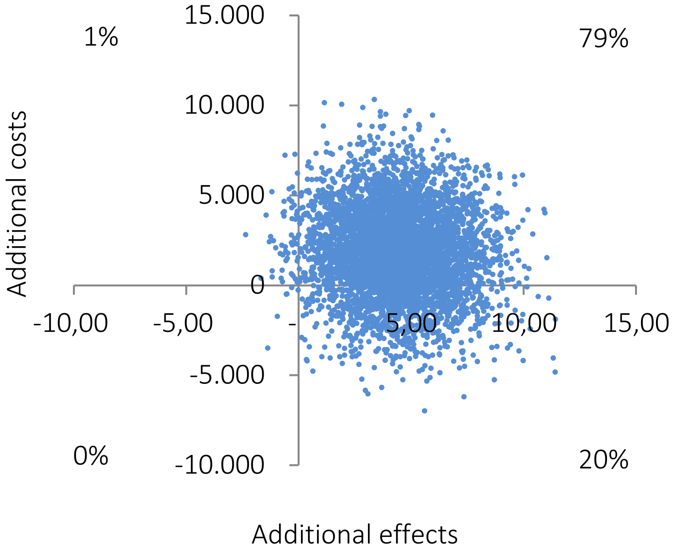

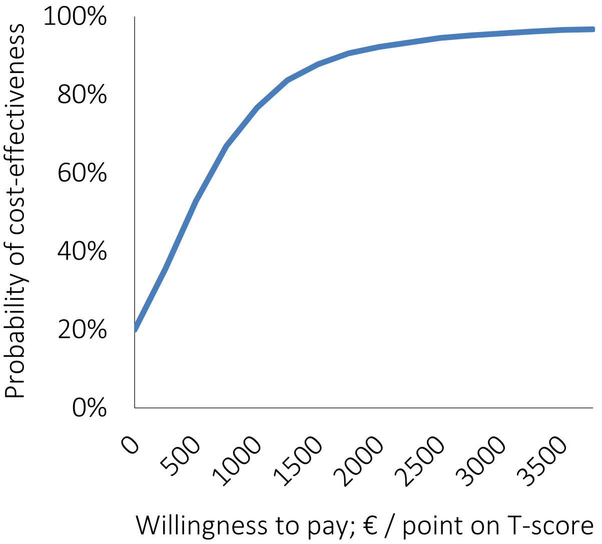

Social care perspective

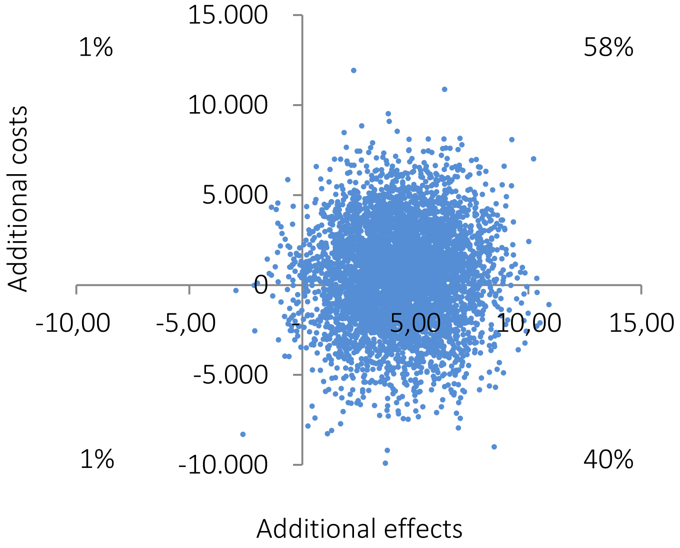

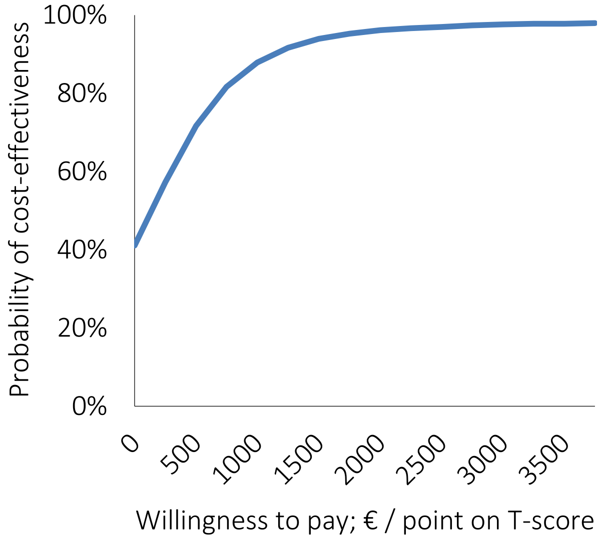

Societal perspective

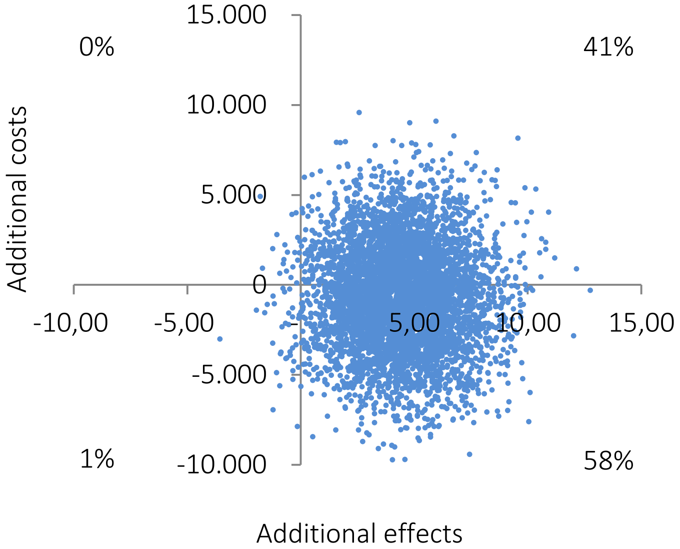

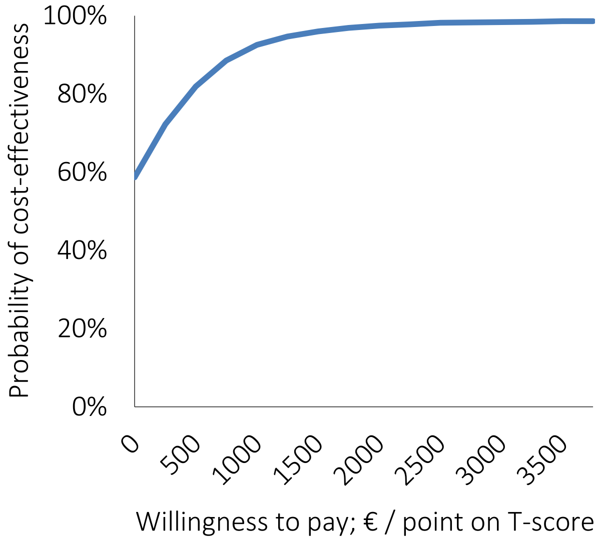

Alternative scenario C

Healthcare perspective

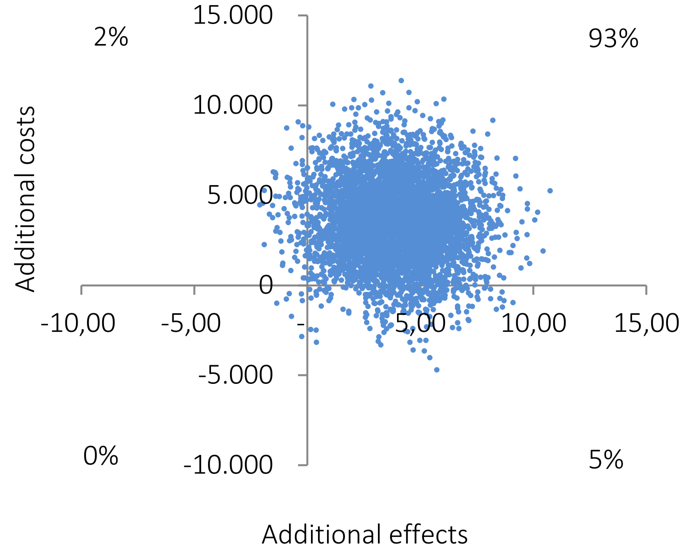

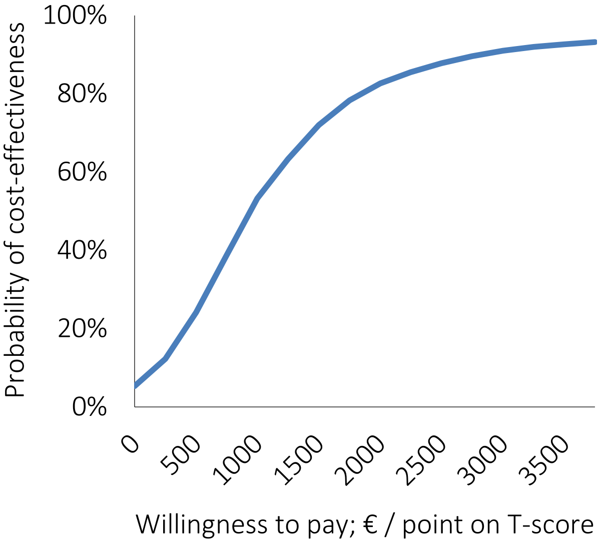

Social care perspective

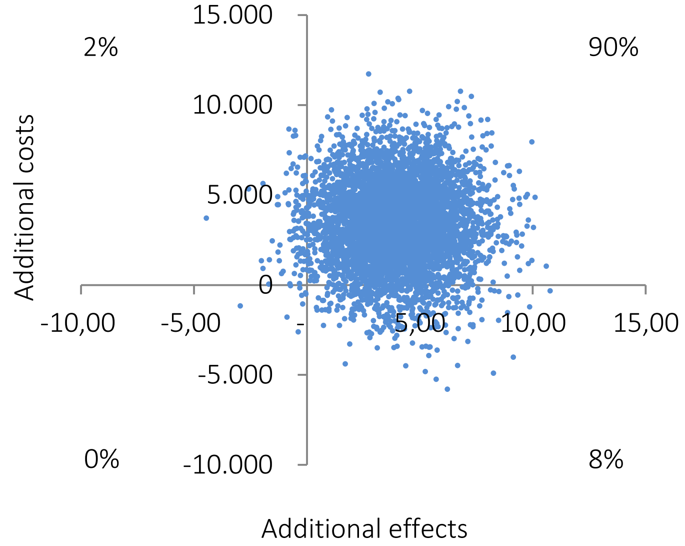

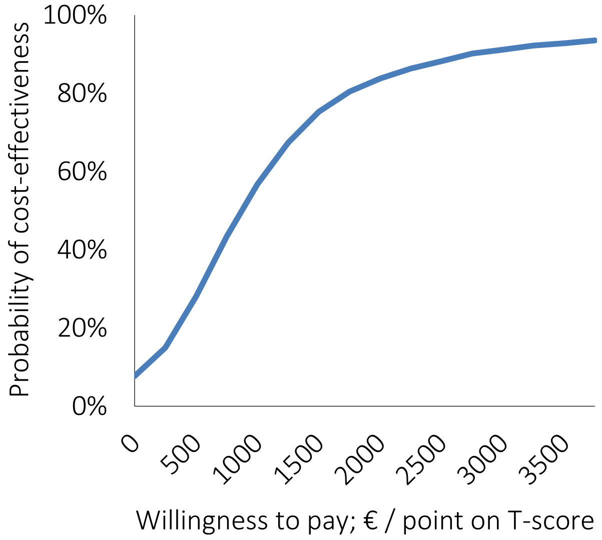

Societal perspective

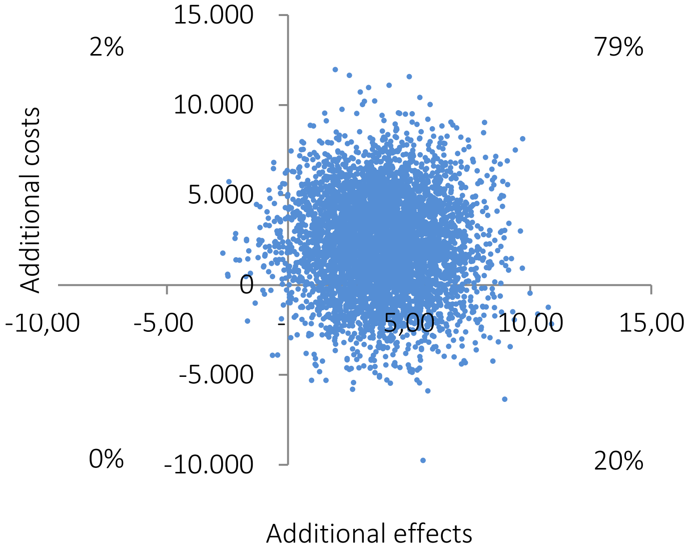

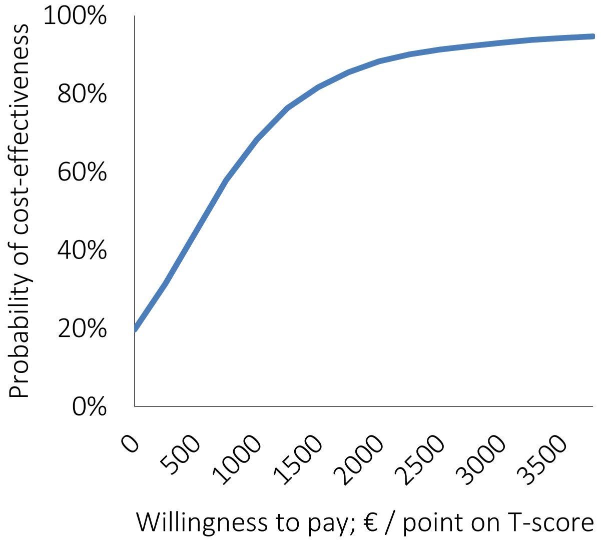

Alternative scenario D

Healthcare perspective

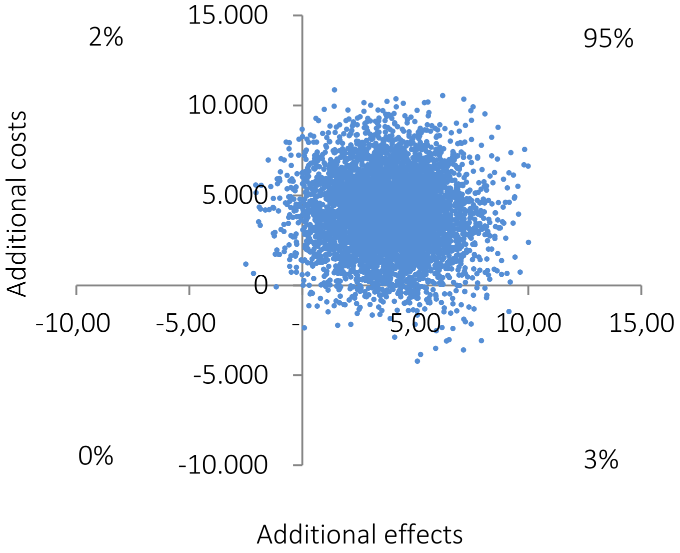

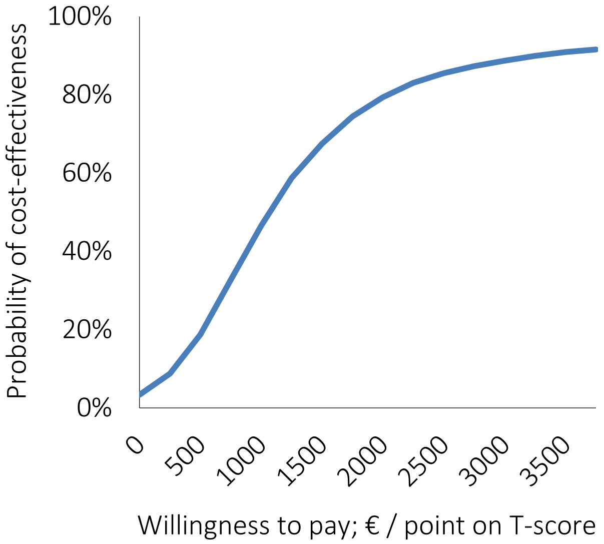

Social care perspective

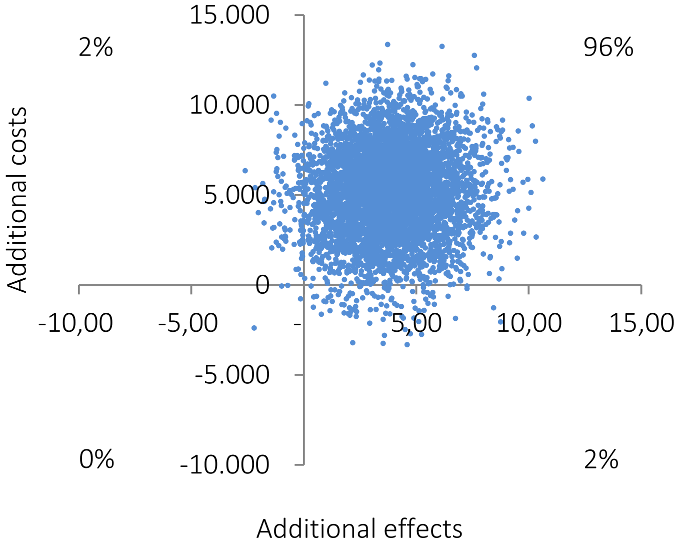

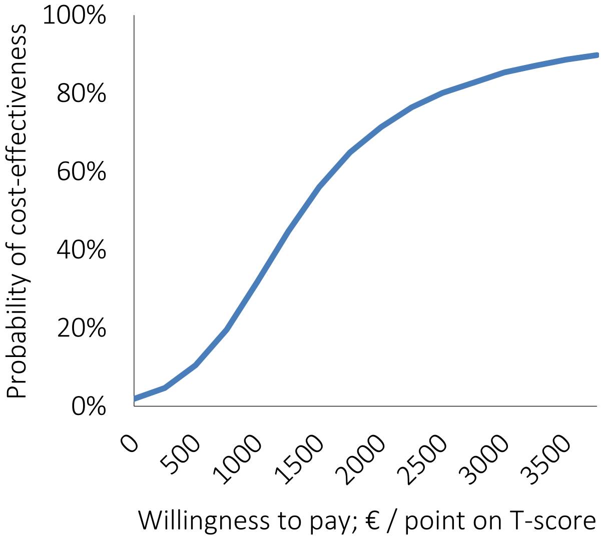

Societal perspective

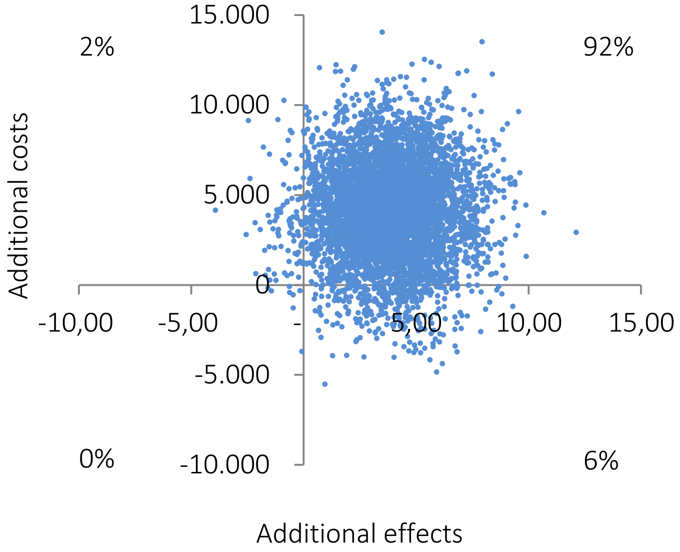

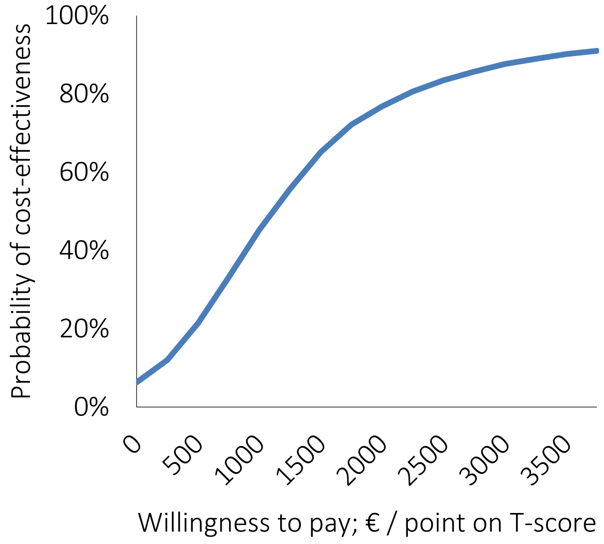

Supplement: Additional file 2: — Cost-effectiveness planes and CEACs for alternative scenarios. This figure shows the scatterplots of simulated incremental cost-effectiveness ratios (n = 5000) on cost-effectiveness planes and CEACs for the PBCM versus the control condition in four alternative scenarios: 1) excluding outliers (alternative scenario A), 2) based on complete cases (alternative scenario B), 3) the sample that actually received the intervention (alternative scenario C) and 4) corrected for baseline cost differences (alternative scenario D). (PDF 3713 kb) [file 12913_2016_1498_MOESM2_ESM.pdf]
